# High burden of childhood tuberculosis in migrants: a retrospective cohort study from the Thailand–Myanmar border

**DOI:** 10.1186/s12879-022-07569-y

**Published:** 2022-07-11

**Authors:** Amy Carroll, Banyar Maung Maung, Win Pa Pa Htun, Wanitda Watthanaworawit, Michele Vincenti-Delmas, Colette Smith, Pam Sonnenberg, Francois Nosten

**Affiliations:** 1 Institute for Global Health, University College London, Mortimer Market Centre, London, WC1E 6JB UK; 2 Shoklo Malaria Research Unit, Mahidol-Oxford Tropical Medicine Research Unit, Faculty of Tropical Medicine, Mahidol University, Mae Sot, Thailand; 3 Centre for Tropical Medicine and Global Health, Nuffield Department of Medicine Research Building, University of Oxford Old Road Campus, Oxford, UK

**Keywords:** Childhood, Tuberculosis, Migrant, Thailand–Myanmar border

## Abstract

**Background:**

Tuberculosis (TB) is a leading cause of morbidity and mortality in children but epidemiological data are scarce, particularly for hard-to-reach populations. We aimed to identify the risk factors for unsuccessful outcome and TB mortality in migrant children at a supportive residential TB programme on the Thailand–Myanmar border.

**Methods:**

We conducted retrospective analysis of routine programmatic data for children (aged ≤ 15 years old) with TB diagnosed either clinically or bacteriologically between 2013 and 2018. Treatment outcomes were described and risk factors for unsuccessful outcome and death were identified using multivariable logistic regression.

**Results:**

Childhood TB accounted for a high proportion of all TB diagnoses at this TB programme (398/2304; 17.3%). Bacteriological testing was done on a quarter (24.9%) of the cohort and most children were diagnosed on clinical grounds (94.0%). Among those enrolled on treatment (n = 367), 90.5% completed treatment successfully. Unsuccessful treatment outcomes occurred in 42/398 (10.6%) children, comprising 26 (6.5%) lost to follow-up, one (0.3%) treatment failure and 15 (3.8%) deaths. In multivariable analysis, extra-pulmonary TB [adjusted OR (aOR) 3.56 (95% CI 1.12–10.98)], bacteriologically confirmed TB [aOR 6.07 (1.68–21.92)] and unknown HIV status [aOR 42.29 (10.00–178.78)] were independent risk factors for unsuccessful outcome. HIV-positive status [aOR 5.95 (1.67–21.22)] and bacteriological confirmation [aOR 9.31 (1.97–44.03)] were risk factors for death in the secondary analysis.

**Conclusions:**

Children bear a substantial burden of TB disease within this migrant population. Treatment success rate exceeded the WHO End TB target of 90%, suggesting that similar vulnerable populations could benefit from the enhanced social support offered by this TB programme, but better child-friendly diagnostics are needed to improve the quality of diagnoses.

## Background

Infection with *Mycobacterium tuberculosis* has the propensity to progress rapidly to tuberculosis (TB) disease and severe or disseminated forms in children. TB is notoriously difficult to diagnose in children, which can lead to TB being overlooked and diagnoses missed [1]. Conversely, inconsistent diagnostic criteria and investigations can lead to overreporting in some instances. Overall, notification data appear to vastly understate the global TB burden among children: half a million children with TB were notified in 2019, however modelling studies have estimated that double the number of children develop tuberculosis annually [1, 2]. Improving the quality of notification data for children and adolescents has been highlighted as a priority by the World Health Organisation (WHO) and is key to understanding the TB burden among children in resource-poor settings and inform programmatic action.

Thailand and Myanmar, two neighbouring countries in Southeast Asia, are among the thirty high-burden TB countries globally as defined by the WHO [1]. Tak province in the Northwest of Thailand is a regional economic hub bordering with Myanmar. The border is porous, and many thousands of Myanmar migrants work within the formal or informal sectors in Thailand’s border towns and rural agricultural industries. Cross-border migrants and their families lack access to effective healthcare, resulting in large health disparities between migrants and native Thais [3, 4]. We have previously reported TB treatment outcomes in the adult migrant population; a group disproportionately burdened by TB and human-immunodeficiency virus (HIV), but there is a lack of patient-level data on paediatric TB [5]. Children are exposed to TB primarily through contact with infectious adults with risk amplified in high TB/HIV settings [6, 7]. Timely diagnosis and treatment is crucial to interrupt transmission and reduce TB-related mortality among vulnerable children. In this study, we sought to identify the characteristics, treatment outcomes and risk factors associated with unsuccessful outcome and mortality in migrant children with TB over a 6-year period.

## Methods

### Setting

The TB programme is run by Shoklo Malaria Research Unit (SMRU), a non-governmental organisation and field research unit linked to the Mahidol-Oxford Tropical Network. It was established to provide care for Myanmar migrants who are not eligible for free healthcare from Thai government hospitals or required additional supportive services. SMRU has developed a residential TB programme where patients stay at the treatment centre for the duration of treatment, with the goal of reducing the financial burden of TB treatment and facilitating treatment adherence [4]. The two treatment centres are located rurally on either side of border: Kou Ko clinic in Myanmar, Wang Pha clinic in Thailand. Patients self-presented to these clinics or were referred from Mae Tao Clinic (Thailand) which is run by a separate organisation. Active case finding is through screening of healthcare workers, household contacts of people with TB and people living with HIV identified through prevention of mother-to-child transmission programme. The TB villages provide a comprehensive package of care for children and their care-givers. Medication, meals, psychosocial support and health education are provided free-of-charge. Recent costing analysis showed that this intervention was highly cost-effective, achieving an estimated cost of GBP 73.4 per DALY averted (W.P.P Htun, personal communication, Apr 5 2022).

### Study design

**Fig. 1:**
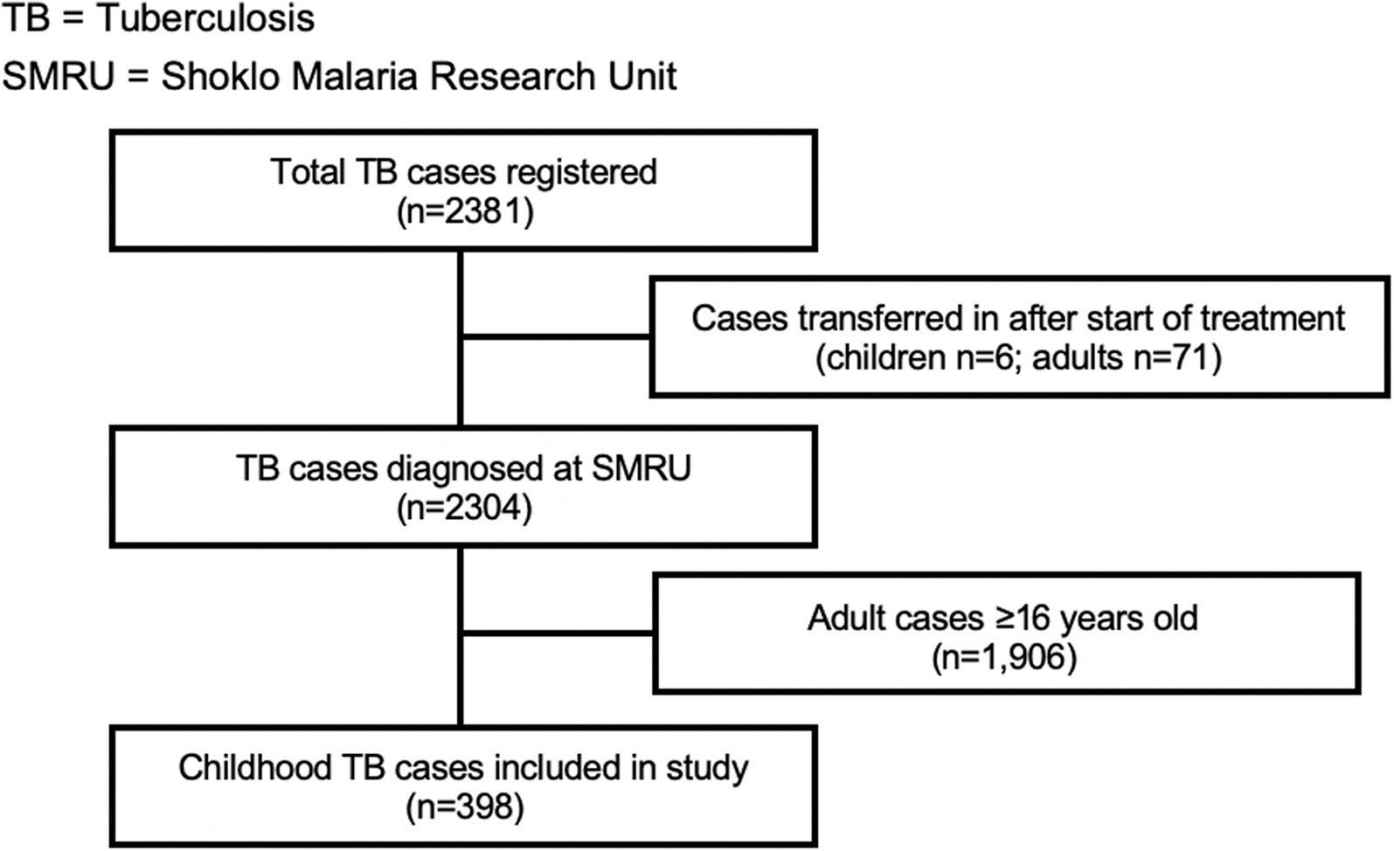
Study population for cohort 2013–2018

Children with TB were identified from a register on which routine programmatic data was recorded for all people with TB between January 2013-December 2018 (n = 2381). We included children aged ≤ 15 years old (n = 398) in this retrospective cohort study. Children that were transferred in following treatment initiation (n = 6) were excluded due to potential survival bias in this group (Fig. 1). TB was diagnosed either bacteriologically via microscopy, culture or Xpert MTB/RIF, or clinically, based on suggestive history, symptoms, exposure and chest X-ray imaging with negative or absent bacteriological testing. Chest X-ray was done on initial assessment for 88.9% of children (n = 354), of whom a substantial proportion (97.2%, n = 344) were documented to have an abnormal X-ray consistent with a diagnosis of TB. HIV tests were done at the start of TB treatment for the majority of children enrolled (98.9%). Only the first TB episode per child in the study period was considered (one child had a second relapse TB episode 6 months after initial treatment completion).

Children were classified as having either extra-pulmonary TB (EPTB) or pulmonary TB, the latter of which included children with both pulmonary and EPTB. ‘New’ TB was defined as TB disease with no prior treatment or less than 1 month of previous TB treatment. Treatment outcomes were defined using standard WHO criteria for children diagnosed bacteriologically [8]. Treatment response in children diagnosed on clinical grounds was assessed through regular clinical review, with treatment failure defined as a lack of response to 2 months of TB treatment, evidenced by persistence of symptoms, failure to thrive or progression of radiological changes [8]. Children enrolled into treatment were initiated on a TB treatment regimen per local guidelines, based on WHO recommendations (2010) [9].

Body weight was measured at the start of TB treatment and at month 2, 3, 5, 6 and 8 (if continuing on a longer course of treatment). Weight-for-age z-scores at the start of treatment were calculated for children whose initial weight was documented. Children were categorized as malnourished if their z-score was below minus two (− 2.0) standard deviations (SD) below the reference mean. Detailed data on age in months was missing for a large proportion of the cohort (297/398). For the purpose of analysis, children with age documented as zero were adjusted to 6 months of age to avoid skewing z-scores. Median weight gain was calculated at month 2 (n = 293) and month 6 (n = 285), where data were available. Month 5 weight was used where month 6 weight was not documented (n = 46).

### Analysis

The primary outcome was unsuccessful treatment outcome (defined as treatment failure, loss to follow up and death) with a secondary analysis for death alone. We report the outcomes for all children diagnosed with TB (n = 398) alongside treatment outcomes for children enrolled in treatment (n = 367). By way of definition, children who were cured or completed treatment were classified as a successful outcome, while children who died, were lost to follow up or failed treatment were classified as having unsuccessful treatment outcome. Children that were transferred out to other TB facilities were classified as not evaluated and were excluded from statistical analysis. We report the frequency and percentage of baseline demographic and clinical characteristics in the cohort. Risk factors for unsuccessful outcome and for death (compared to successful outcome) were identified with logistic regression. Hypothesis testing was done using χ^2^ or Fishers exact test for each clinical variable. The logistic regression models included age and gender a priori and a broad inclusion criterion of p ≤ 0.20 (χ^2^ or Fishers exact) was used for selection of clinical variables into the model. Variables with zero children in one of the comparator groups were excluded from the logistic regression models. Interaction terms were used to check for effect modification. Odds ratios (ORs), adjusted ORs (aORs) and corresponding 95% confidence intervals (CIs) were used to quantify differences between groups; p-value < 0.05 was considered significant. Statistical analysis was performed using STATA/IC 14.2.

The analysis used routinely collected anonymised programmatic data. The research was operationally necessary with the aim of reviewing services and informing resource allocation. Ethical approval was obtained from the Oxford Tropical Research Ethics Committee (reference 554-20) and the need for informed consent was waived due to the retrospective nature of the study.

## Results

### Study population and treatment outcomes

Between January 2013 and December 2018, a total of 2304 people were diagnosed with TB across the Shoklo Malaria Research Unit sites. Of these, there were 398 (17.3%) children diagnosed with TB, and 367 of these were enrolled in treatment (Fig. 1). A greater proportion of TB was diagnosed in males (57.0%) and among children aged 0–4 years old (55.0%). Ninety nine children were aged below 2 years old at diagnosis. The majority of children had pulmonary TB (93.5%), including four children with both pulmonary and extra-pulmonary disease (TB meningitis, abdominal, lymph node, spinal). The remaining 6.5% of children had extra-pulmonary TB: nine TB meningitis, three abdominal, three lymph node, two pleural, one spinal and eight with EPTB at undocumented sites. The majority of children with TB were clinically diagnosed (94.0%). Bacteriological testing consisting of one of (or a combination of) smear microscopy, Xpert testing or TB culture of the relevant sample was done in 99/398 children (24.9%). TB disease was confirmed in 24/99 (24.2%). The majority of children who had samples tested were in the older age group (n = 87, 21/87 positive). Twelve children aged 0–4 had samples taken for testing, of which three confirmed TB. Among those bacteriologically confirmed, four children had drug resistance detected on Xpert (two had MDR-TB confirmed on culture, and two did not have TB culture done but had an MDR-TB contact), all four were treated as multi-drug resistant TB (MDR-TB) (Table 1). No children had mono-drug resistant TB. Mean weight for age z-score was − 2.2 (SD 1.3), indicating that the majority of children met criteria for being malnourished at the time of diagnosis. A total of 9.8% (39/398) of the cohort were HIV-positive (twenty aged 0–4 years, nineteen aged 5–15 years), of whom most were new diagnoses (30/39) (Table 1). The majority of HIV-positive children were started on anti-retroviral therapy (ART) (31/39); of the remaining children, five were transferred out prior to starting TB/ART treatment and three died early during TB treatment prior to starting ART. HIV status was unknown in 24/398 children, but was documented in the majority enrolled in treatment (98.9%).

**Table 1:** Demographic and clinical characteristics of 398 children diagnosed with TB and their treatment outcomes

Characteristic	Variable	Total N = 398	Successful outcome* N = 332	Unsuccessful outcome* N = 42
		Number (%) unless otherwise indicated		
Gender	Male	227 (57.0)	193 (58.1)	19 (45.2)
	Female	171 (43.0)	139 (41.9)	23 (54.8)
Age	0–4	219 (55.0)	185 (55.7)	21 (50.0)
	5–15	179 (45.0)	147 (44.3)	21 (50.0)
Mean weight-for-age z-score (SD; n) ^†^		− 2.2 (1.3; n = 300)	− 2.2 (1.3; n = 283)	− 1.9 (1.8; n = 17)
Year diagnosed	2013–2015	199 (50.0)	175 (52.7)	19 (45.2)
	2016–2018	199 (50.0)	157 (47.3)	23 (54.8)
TB site	Pulmonary^‡^	372 (93.5)	316 (95.2)	35 (83.3)
	Extra-pulmonary	26 (6.5)	16 (4.8)	7 (16.7)
TB history	New	357 (89.7)	322 (97.0)	28 (66.7)
	Retreatment	13 (3.3)	10 (3.0)	2 (4.7)
	No data	28 (7.0)	0 (0.0)	12 (28.6)
Bacteriologic confirmation	Negative/Not done	374 (94.0)	321 (96.7)	34 (81.0)
	Positive	24 (6.0)	11 (3.3)	8 (19.0)
Drug susceptibility	Non-MDR-TB	394 (99.0)	330 (99.4)	40 (95.2)
	MDR-TB	4 (1.0)	2 (0.6)	2 (4.8)
Treatment regimen	Category I	352 (88.4)	322 (97.0)	26 (61.9)
	Non-Category I	15 (3.8)	10 (3.0)	4 (9.5)
	Not started^§^	31 (7.8)	0 (0.0)	12 (28.6)
HIV status	Negative	335 (84.2)	300 (90.4)	26 (61.9)
	Positive^§§^	39 (9.8)	29 (8.7)	5 (11.9)
	Unknown	24 (6.0)	3 (0.9)	11 (26.2)

*TB* tuberculosis, *MDR-TB* multi-drug resistant TB, *HIV* human-immunodeficiency virus

*Comparison between groups with successful and unsuccessful outcomes excludes 24 children transferred to other care providers either before or after starting treatment, whose treatment outcome was not evaluated

^†^At start of TB treatment, where data available

^‡^Pulmonary TB category includes four children who had both pulmonary and extra-pulmonary TB disease

^§^Treatment not started in 19 children that were transferred out, 11 that were lost to follow-up and 1 that died before starting TB treatment

^§§^HIV positive children include 8 children not on antiretroviral therapy (3 died early during TB treatment, 5 transferred out)

Treatment outcomes were evaluated; the treatment success rate was high among those that started treatment (332/367; 90.5%) (Table 2). Among all children diagnosed with TB, losses to follow-up were low (26/398; 6.5%). Of these, eleven children were lost to follow-up pre-treatment and fifteen children were lost to follow-up after treatment initiation. The case fatality rate was 3.8% (95% CI 2.1–6.1%), equating to a total of 15 deaths, with one death prior to treatment initiation.

**Table 2:** Treatment outcomes of children diagnosed with TB and of children started on TB treatment at Shoklo Malaria Research Unit, 2013–2018

Outcome		All children diagnosed with TB (n = 398)	Children started on treatment (n = 367)
		Number (%) unless otherwise indicated	
Successful	Cure/Complete	332 (83.4)	332 (90.5)
Unsuccessful	Total	42 (10.6)	30 (8.2)
	Died	15 (3.8)	14 (3.8)
	Treatment failure	1 (0.3)	1 (0.3)
	Loss to follow-up	26 (6.5)	15 (4.1)
Not evaluated*		24 (6.0)	5 (1.3)
Median weight gain, kg (IQR)	2 months (n = 293)	–	1 (0–1.5)
	6 months (n = 285)	–	2 (1–3)

*Not evaluated: 24 children transferred out to other care providers either prior to starting TB treatment or following treatment initiation

TB = tuberculosis

### Risk factors for unsuccessful outcome and mortality

Risk factors were identified using logistic regression. In univariable analysis EPTB, bacteriologically positive TB, MDR-TB and unknown HIV status were risk factors for unsuccessful outcome. In the multivariable model, children with EPTB (aOR 3.56, CI 1.12–10.98), bacteriologically confirmed TB (aOR 6.07, CI 1.68–21.92) and unknown HIV status (aOR 42.29, CI 10.00–178.78) had significantly increased odds of unsuccessful treatment outcome, compared to those with pulmonary TB, negative/absent bacteriological tests and HIV negative status respectively (Table 3).

**Table 3:** Logistic regression analysis showing risk factors for unsuccessful treatment outcome and death versus successful outcome (n = 332) among children diagnosed with TB

	Unsuccessful outcome (total n = 374; 42 unsuccessful outcomes vs 332 successful outcomes)*					Death (total n = 347; 15 deaths vs 332 successful outcomes)^†^				
	n (%)	Crude		Adjusted		n (%)	Crude		Adjusted	
		OR (95% CI)	p	OR (95% CI)	p		OR (95% CI)	p	OR (95% CI)	p
Sex										
Male	19 (45.2)	Ref.	–	–	–	7 (46.7)	Ref.	–	–	–
Female	23 (54.8)	1.68 (0.89–3.21)	0.12	1.12 (0.52–2.43)	0.77	8 (53.3)	1.59 (0.56–4.48)	0.38	1.37 (0.43–4.29)	0.60
Age, years										
0–4	21 (50.0)	Ref.	–	–	–	9 (60.0)	Ref.	–	Ref.	–
5–15	21 (50.0)	1.26 (0.66–2.39)	0.41	1.09 (0.51–2.32)	0.83	6 (40.0)	0.84 (0.29–2.41)	0.74	0.65 (0.20–2.12)	0.47
TB site										
PTB	35 (83.3)	Ref.	–	–	–	12 (80.0)	Ref.	–	–	–
EPTB	7 (16.4)	3.95 (1.52–10.26)	0.005	3.56 (1.12–10.98)	0.03	3 (20.0)	4.94 (1.27–19.26)	0.02	4.26 (0.91–20.61)	0.07
TB history										
New	28 (66.7)	Ref.	–	–	–	12 (80.0)	Ref.	–	–	–
Retreatment	2 (4.7)	2.30 (0.48–11.02)	0.30	–	–	2 (13.3)	5.37 (1.06–27.22)	0.04	–	–
No data^‡^	12 (28.6)	–	–	–	–	1 (6.7)	–	–	–	–
Bacteriologically +										
Negative/No data	34 (80.1)	Ref.	–	–	–	11 (73.3)	Ref.	–	–	–
Yes	8 (19.0)	6.87 (2.58–18.24)	< 0.001	6.07 (1.68–21.92)	0.01	4 (26.7)	10.61 (2.91–38.65)	< 0.001	9.31 (1.97–44.03)	0.005
MDR-TB^§^										
No	40 (95.2)	Ref.	–	–	–	15 (100)	–	–	–	–
Yes	2 (4.8)	8.25 (1.13–60.19)	0.04	0.31 (0.01–6.65)	0.45	0	–	–	–	–
HIV status										
Negative	26 (61.9)	Ref.	–	–	–	9 (60.0)	Ref.	–	–	–
Positive	5 (11.9)	1.98 (0.71–5.57)	0.19	2.07 (0.69–6.15)	0.19	5 (33.3)	5.74 (1.81–18.29)	0.003	5.95 (1.67–21.22)	0.006
Unknown	11 (26.2)	42.31 (11.10–161.25)	< 0.001	42.29 (10.00–178.78)	< 0.001	1 (6.7)	11.11 (1.05–117.48)	0.05	7.31 (0.59–90.00)	0.12

*TB* tuberculosis, *MDR-TB* multi-drug resistant TB, *HIV* human-immunodeficiency virus, *OR* odds ratio, *CI* confidence interval

*Reference group excludes children transferred out (n = 24)

^†^Reference group excludes children with unknown mortality status (loss to follow up (n = 26), transfer out (n = 24)) and treatment failure (n = 1)

^‡^TB history: variable ‘No data’ excluded from logistic analysis as zero children with no data in comparator group

^§^MDR-TB excluded from logistic analysis for death as zero children in group

Risk factors for mortality were assessed separately. The reference group for this analysis included children with successful outcome (n = 332) and excluded children whose mortality status was unknown (those with outcomes of lost to follow up or transfer out) and the one child with treatment failure. In univariable analysis EPTB, bacteriological confirmation, TB retreatment and positive or unknown HIV status were associated with death. Independent risk factors for mortality were HIV positivity (aOR 5.95, CI 1.67–21.22) and bacteriologically confirmed TB (aOR 9.31, CI 1.97–44.03) compared to HIV negative children and those with negative/absent bacteriological tests respectively. In addition, there remained a trend towards higher odds of death among those with EPTB. We were unable to evaluate the multivariable associations of MDR-TB and TB history with death due to small numbers in some strata (Table 3).

## Discussion

This retrospective cohort study reports treatment outcomes and risk factors for unsuccessful outcome and death among migrant children within a TB programme at the Thailand–Myanmar border over a 6-year period (2013–2018). In this study, children aged ≤ 15 years old constituted 17.3% of the overall TB burden within this TB programme. Among children enrolled in treatment, nine out of ten had a successful treatment outcome. EPTB, bacteriological TB confirmation and unknown HIV status were risk factors for unsuccessful outcome (defined as loss to follow-up, treatment failure or death). The case fatality rate was 3.8% and HIV positivity and bacteriological confirmation of TB were risk factors for mortality. The vulnerability of this population was highlighted in our previous study which focused on the adult cohort. In comparison to this cohort of children, we found that a lower proportion of adults completed treatment successfully (79.8%), and losses to follow-up and case fatality were substantially higher (9.0% and 9.5% respectively) [5].

The proportion of overall TB diagnoses in children (17.3%) was higher than observed in other studies within Thailand and other TB endemic countries (2–9%) and exceeds the WHO global estimate of 11% [1, 10–12]. Globally, childhood TB is consistently under-reported, catalysing increased advocacy and commitment towards improving case-detection rates [13]. There are several explanations for the high proportion of children with TB observed in our study. Firstly, there is a high double burden of TB and HIV in this population, as demonstrated in our previous study of adults in this programme [5]. Thailand has a generalised HIV epidemic and HIV prevalence among migrant workers is estimated to be up to four times higher than among the general population [14]. Children, particularly infants, living in HIV-affected households are at increased risk of TB exposure and infection irrespective of their own HIV status [7, 15]. Additionally, children living in crowded households with a person with infectious TB are likely to experience prolonged and high-intensity exposure to TB, increasing their risk of TB infection and progression to active disease [16]. These factors could partially explain the high burden of TB disease observed among children in this population, and highlight the importance of continued active case-finding for household contacts of adults with TB, symptom-based screening and tuberculosis preventive therapy (TPT) provision for children [13]. At this programme, TPT is currently provided for children under 5 years old and household contacts of people with TB/HIV, however this may be adapted with updated guidelines to provide TPT to children and adolescents under 18 years old. An alternative explanation for the high proportion of children with TB observed in this study could be the population age structure of migrants along the Thailand–Myanmar border. Accurate estimates of the numbers of child migrants in this region are lacking (enumeration is challenging given migrants’ irregular legal status), however the age structure of immigrant populations in Asia are skewed towards children and young adults which could result in a higher than expected burden of disease [17].

The majority of children had TB diagnosed on clinical grounds (94.0%) and were treated empirically in absence of confirmatory diagnostic tests. There was a low proportion of children with bacteriologically diagnosed TB (6.0%) and EPTB (6.5%) in our cohort, compared to studies in Thailand and other TB endemic countries [10–12, 18, 19]. This could imply an element of over-diagnosis, however in our cohort only a quarter had samples taken for bacteriological testing which limits our ability to draw strong conclusions about this. The challenges of diagnosing TB in children are myriad and magnified in resource-poor settings. Firstly, obtaining an appropriate sample from children is resource-intensive. Studies have shown that diagnostic yield can be increased substantially with induced sputum and when a second specimen is taken [20], however, this is rarely feasible in low resource settings. Secondly, the paucibaccillary nature of TB in young children means that the diagnostic yield of traditional smear and culture are very low (< 20%). Molecular testing of either sputum or stool specimens from children with clinically suspected TB on Xpert MTB/RIF increases sensitivity and the newer Xpert MTB/RIF Ultra has even higher diagnostic sensitivity. Molecular testing on stool is now recommended by the WHO and is a low cost, non-invasive tool which could increase bacteriologic testing in low resource settings like ours [21].

A further challenge that is particularly salient in our population is differentiating TB from other common childhood illnesses with clinically overlapping signs, particularly in the most vulnerable groups such as those with HIV, malnutrition or chronic lung conditions. Diagnostic scoring systems have been shown to perform poorly in settings with a high burden of these comorbid conditions [22]. Empirical treatment based on the available clinical evidence is important and life-saving [23]. A quarter of our cohort were less than 2 years of age; in light of the high prevalence of malnutrition and TB, it might be reasonable for clinicians to have a lower threshold to starting TB treatment. Our results highlight the need for child-friendly diagnostic tests which can be used at the point of care to accurately identify children with TB and avoid diagnostic delays and unnecessary TB treatment in instances where the diagnosis in unclear.

This programme achieved a treatment success rate of 90.5% among children enrolled in treatment, meeting the End TB target of 90% and weight gain was demonstrated at the 2 and 6 months marks. Unsuccessful outcomes were seen in one-tenth of children, with the majority of these consisting loss to follow-up (26/42 children, 6.5% overall). The vulnerability of migrant children was highlighted in Lolekha et al. surveillance study of child TB outcomes across four Thai provinces and one national hospital, which found very high rates of loss to follow-up in non-Thai children (48%), who were six times more likely to be lost to follow-up than children of Thai nationality [10]. Qualitative studies by Tschirhart et al. highlighted the multiple barriers adult migrants at the Thailand–Myanmar border face when accessing TB/HIV care, including lack of information about services, traveling long distances to facilities and living in unstable accommodation. Parents’ fragile work arrangements make children with TB particularly vulnerable to treatment abandonment [4, 5, 24, 25]. At this TB programme, standard care is supplemented with free accommodation, regular meals and group psychosocial and educational support. Services have been streamlined so that wherever possible, TB treatment is initiated on the same day as presentation if clinical suspicion is high in order to engage children and their care-givers early in the treatment process. Our results suggest that this TB programme addresses the gap in TB care for this mobile population and provides the support and tools needed to maintain children on TB treatment. Future qualitative research focusing specifically on the experiences of children and care-givers when accessing TB care could provide more information on how to further improve and strengthen services.

Unfortunately, these interventions do not always mitigate the drivers of non-adherence and loss to follow-up [26, 27]. In our study two of the four children with MDR-TB were lost to follow-up and children with markers of clinically advanced or highly infective TB disease were at the greatest risk of unsuccessful outcome, including death and loss to follow-up. Long, potentially toxic treatment regimens often contribute to non-adherence and loss to follow-up. The WHO have recently approved a 4-month TB drug regimen for children and adolescents with non-severe, smear-negative, presumed drug-susceptible TB after the SHINE trial showed non-inferiority when compared to the standard 6-month regimen [28]. The trial involved participants in Africa and India and 11% of the cohort had TB/HIV coinfection, similar to our cohort. Children with confirmed drug resistance or with known exposure to an adult with any drug-resistant TB were excluded. This could be potential barrier to its introduction in our setting due the high rates of drug-resistance [1, 5]. Shorter regimens could reduce the burden on families and programme costs but more detail is needed on how to determine eligibility.

Children living with HIV are at increased risk of TB disease, developing more severe forms of TB and TB-related death [7]. In our study, 9.8% of children had TB/HIV coinfection—lower than observed in the adult population (18.5%) and other studies among children in Asian and African countries where rates of TB/HIV ranged from 10 to 60% [5, 7, 10–12] In our cohort, the majority of children with HIV were new diagnoses, suggesting that in many cases children presented with advanced immune-suppression, though further evaluation is limited by a lack of data on lymphocyte subsets or viral loads. The strongest predictors of death among children in our study were HIV infection and bacteriological confirmation of TB, consistent with other studies [10–12] Continued cross-border collaborative planning and information-sharing around TB, TB/HIV and tuberculosis preventive therapy for migrant populations is essential to strengthen services and maintain children within these services.

Our study has several limitations. It is retrospective therefore the accuracy of clinical diagnosis cannot be commented. Bacteriologic testing rates were low (a quarter of the cohort) thus limiting the strength of conclusions that can be drawn from multivariable analysis. Positive bacteriological testing was strongly associated with poor outcomes, which likely reflects that the most unwell children were prioritised for collecting samples. There were small numbers in some sub-categories and results with wide CIs should be interpreted with caution. Children were classified as having MDR-TB only when drug resistance was confirmed on culture and/or GeneXpert and no children were treated presumptively therefore the burden of MDR-TB may be under-estimated. We acknowledge that in some cases z-score may have been over or under-estimated for young children where detailed age data (months of age) was missing. Data were not available for patient-level characteristics that may be associated with poor outcomes such as HIV staging, CD4 counts and viral loads. We also lack detailed information on symptom history and specific causes of death. Our study’s primary strengths are that it reports high rates of diagnosis and treatment success among a very vulnerable population of children. It is the only study that we are aware of that describes TB epidemiology in migrant children in low or middle-income countries and our study describes specific interventions that have been highly effective at maintaining children on TB treatment in this setting.

## Conclusion

We found that children bear a substantial burden TB within this migrant population. Due to scarce resources, there was limited bacteriologic testing and a very high proportion of children were treated empirically. Accurate TB diagnosis in settings where there is a high burden of childhood comorbidities is challenging and our study highlights the need for roll-out of child-friendly diagnostics suitable for use in low-resource settings, such as molecular testing on stool samples. Our study highlights the importance of stepping up programmatic efforts to identify and treat TB in children from hard-to-reach groups and demonstrates that excellent treatment success rates can be achieved when using supportive care packages to stabilise the living situations of children and their care-givers.

## Data Availability

The datasets generated and/or analysed during the current study are not publicly available in order to prevent deductive disclosure, but a redacted dataset may be available for research and policy purposes on reasonable request. Requests for data should be submitted to the Data Sharing Committee of Mahidol Oxford Tropical Medicine Research Unit via email to francois@tropmedres.ac.

## Abbreviations

TB: Tuberculosis
WHO: World Health Organisation
SMRU: Shoklo Malaria Research Unit
EPTB: Extra-pulmonary TB
SD: Standard deviations
OR: Odds ratios
aOR: Adjusted ORs
CI: Confidence intervals
ART: Anti-retroviral therapy
MDR TB: Multidrug-resistant TB
TPT: Tuberculosis preventive therapy
